# Comprehensive analysis of *SPAG1* expression as a prognostic and predictive biomarker in acute myeloid leukemia by integrative bioinformatics and clinical validation

**DOI:** 10.1186/s12920-022-01193-0

**Published:** 2022-02-28

**Authors:** Yu Gu, Ming-qiang Chu, Zi-jun Xu, Qian Yuan, Ting-juan Zhang, Jiang Lin, Jing-dong Zhou

**Affiliations:** 1 Department of Hematology, Affiliated People’s Hospital of Jiangsu University, 8 Dianli Rd., Zhenjiang, 212002 Jiangsu People’s Republic of China; 2 Laboratory Center, Affiliated People’s Hospital of Jiangsu University, 8 Dianli Rd., Zhenjiang, 212002 Jiangsu People’s Republic of China; 3 Zhenjiang Clinical Research Center of Hematology, Zhenjiang, 212002 Jiangsu People’s Republic of China; 4 The Key Lab of Precision Diagnosis and Treatment in Hematologic Malignancies of Zhenjiang City, Zhenjiang, 212002 Jiangsu People’s Republic of China; 5 Department of Oncology, Affiliated People’s Hospital of Jiangsu University, 8 Dianli Rd., Zhenjiang, 212002 Jiangsu People’s Republic of China

**Keywords:** *SPAG1*, Expression, Prognosis, AML, Bioinformatics

## Abstract

**Background:**

Recently, an increasing number of studies have reported that sperm-associated antigen (SPAG) proteins play crucial roles in solid tumorigenesis, and may serve as potentially helpful biomarkers for cancer diagnosis and prognosis. However, very few studies systematically investigated the expression of *SPAG* family members and their clinical significance in acute myeloid leukemia (AML).

**Methods:**

The expression of *SPAGs* and their prognostic significance in AML were determined by a systematic analysis on data gathered from public databases, and the results were validated in clinical samples.

**Results:**

Using public data, we identified only increased *SPAG1* expression negatively associated with survival in AML by Cox regression (*P* < 0.001) and Kaplan–Meier analysis (*P* < 0.001). The prognostic value of *SPAG1* expression was further confirmed in other independent cohorts. Clinically, higher *SPAG1* expression was significantly correlated with white blood cell counts (*P* = 0.014) and French–American–British (FAB) subtypes (*P* = 0.024). Moreover, higher *SPAG1* expression was more common in + 8 patients (*P* = 0.034), rarely found with t(8;21) (*P* = 0.014), and correlated with *FLT3* (*P* < 0.001) and *DNMT3A* mutations (*P* = 0.001). Despite these associations, multivariate analysis confirmed the independent prognostic value of *SPAG1* expression in AML (*P* < 0.001). Notably, AML patients with higher *SPAG1* expression may benefit from hematopoietic stem cell transplantation (HSCT), whereas patients with lower *SPAG1* expression appeared less likely to benefit. Finally, we further validated that *SPAG1* expression was significantly increased in newly diagnosed AML patients compared with normal controls (*P* < 0.001) and with AML patients who achieved complete remission (*P* < 0.001). Additionally, *SPAG1* expression could act as a potentially helpful biomarker for the diagnosis and prognosis of AML (*P* < 0.001 and = 0.034, respectively).

**Conclusions:**

Our findings demonstrated that *SPAG1* overexpression may serve as an independent prognostic biomarker and may guide the choice between HSCT and chemotherapy in patients with AML.

**Supplementary Information:**

The online version contains supplementary material available at 10.1186/s12920-022-01193-0.

## Introduction

Acute myeloid leukemia (AML) represents a cohort of clonal hematopoietic malignances that originate from myeloid precursors and is a highly heterogeneous disease in terms of molecular, cytogenetic and clinical features [1]. Genetic and molecular abnormalities are closely associated with the leukemogenesis and prognosis of AML [2]. Although mutations in several genes, such as *FLT3*, *CEBPA*, *NPM1*, *TP53*, *RUNX1* and *ASXL1*, have been well established to occur in AML, the current understanding of the molecular mechanisms involved in the development and progression of AML is still limited [3]. Precise risk stratification and prognosis assessment are of great significance in the selection of treatment for AML patients [4]. Therefore, the identification of a series of molecular alterations that can predict the clinical outcomes of AML patients may contribute to the development of AML–specific targeted therapies.

Evidence has proven that cancer–testis (CT) antigens may function in stemness due to their expression during germ cell and embryonic development, which promotes an important oncogenicity effect in cancer cells [5]. To date, a cluster of proteins named sperm-associated antigens (*SPAG*), of which 15 members (*SPAG1*, *SPAG2*/*UAP1*, *SPAG3*/*SPAG8*, *SPAG4*, *SPAG5*, *SPAG6*, *SPAG7*, *SPAG9*, *SPAG10*/*MFGE8*, *SPAG11B*, *SPAG12*/*NHP2L1*, *SPAG13*/*SSFA2*, *SPAG15*/*SPAM1*, *SPAG16* and *SPAG17*) are CT antigens, has been identified [6]. Over the years, ample research has reported the vital role of SPAG proteins, which may function as promising new biomarkers for diagnosis and prognosis in solid tumorigenesis, yet there is a great lack of systematic investigation of SPAG family member expression and clinical evaluation of these proteins in acute myeloid leukemia (AML) [6].

To date, our research is the first to report that *SPAG1* mRNA expression, among *SPAG* family members, is negatively associated with survival in AML. Moreover, the prognostic value of *SPAG1* overexpression in AML was further confirmed by our data. High expression of *SPAG1* mRNA was intrinsically connected to specific genetic (both cellular and molecular levels) abnormalities in AML. Despite these associations, *SPAG1* overexpression could also function independently as a prognostic biomarker in AML, and it may serve as a reference for consolidation therapy selection between chemotherapy and hematopoietic stem cell transplantation (HSCT).

## Materials and methods

### Public datasets

The identification cohort comprised 173 AML patients with RNA-Seq V2 data for *SPAG* family members (*SPAG1*, *SPAG2*/*UAP1*, *SPAG3*/*SPAG8*, *SPAG4*, *SPAG5*, *SPAG6*, *SPAG7*, *SPAG9*, *SPAG10*/*MFGE8*, *SPAG11B*, *SPAG12*/*NHP2L1*, *SPAG13*/*SSFA2*, *SPAG15*/*SPAM1*, *SPAG16* and *SPAG17*) from The Cancer Genome Atlas (TCGA) [7]. The treatment regimens for these patients included induction therapy and consolidation therapy. All patients received standard chemotherapy as induction therapy. Following induction chemotherapy, a total of 100 patients underwent chemotherapy only, whereas 73 patients received HSCT as consolidation treatment. In addition, the expression of *SPAG1* in AML compared with controls was analyzed in GEPIA (http://gepia.cancer-pku.cn/).

Three independent cohorts from the Gene Expression Omnibus (GEO) database (GSE12417, GSE6891 and GSE37642) were used to validate the prognostic value of *SPAG1* expression in AML. Moreover, the association of the *SPAG1* expression level with the prognosis of *SPAG1* expression level on prognosis of 78 and 162 cytogenetically normal AML (CN-AML) patients was analyzed in the GSE12417 dataset with the public platform GenomicScape (http://genomicscape.com/microarray/survival.php) [8, 9]. The GSE6891 dataset consisted of 461 AML patients, whereas the GSE37642 dataset comprised 562 AML patients. Kaplan–Meier analysis was performed to explore the prognostic value of *SPAG1* expression in two groups with median level of *SPAG1* expression as the cutoff.

### Patients

The validation cohort included 131 AML patients, with 86 enrolled at diagnosis and 45 at complete remission (CR), treated at our hospital. Patients with antecedent hematological diseases or therapy-related AML were eliminated. The clinical characteristics of the cases are presented in Additional file 1: Table S1. Fifteen healthy bone marrow donors served as the controls. The age of the newly diagnosed AML patients (median 52, range 18–81) was similar to that of the AML patients at CR (controls) (median 45, range 28–66). The diagnosis and classification of AML patients followed the 2016 revised World Health Organization (WHO) and French–American–British (FAB) criteria [3, 10]. The treatment regimens of these AML cases were as reported [11–13]. The study protocol was approved by the Institutional Ethics Committee of the Affiliated People’s Hospital of Jiangsu University, and all the volunteers provided written informed consent.

### Sample preparation, RNA isolation and reverse transcription

Clinical bone marrow (BM) specimens were sampled from the validation cohort of AML cases and controls who were treated in our hospital. We separated BM mononuclear cells (BMMNCs) and then extracted total RNA by using Lymphocyte Separation Medium (Solarbio, Beijing, China) and TRIzol reagent (Invitrogen, Carlsbad, CA), respectively. cDNA was synthesized via RNA reverse transcription as described previously [11–13].

### Real-time quantitative PCR (RT–qPCR)

Quantized data of *SPAG1* and *ABL1* (housekeeping gene) transcripts were unfolded by RT–qPCR via AceQ qPCR SYBR Green Master Mix (Vazyme Biotech Co., Piscataway, NJ). The primer sequences were 5′-TCTTCTGCGTCGTGCTAC-3′ (forward) and 5′-TTATCTCCACCGCCATCT-3′ (reverse) for *SPAG1* as well as 5′-TCCTCCAGCTGTTATCTGGAAGA-3′ (forward) and 5′-TCCAACGAGCGGCTTCAC-3′ (reverse) for *ABL1*. The relative *SPAG1* transcript level was calculated based on the 2^−∆∆Ct^ method [11–13].

### Bioinformatics analysis

All procedures referring to bioinformatics analysis were conducted as our previous reports [14, 15]. To obtain the differentially expressed genes/miRNAs (DEGs), RNA-sequencing (mRNA and microRNA) data analysis was performed according to the raw read counts with the R/Bioconductor package “edgeR” based on the filter condition: |log2 fold change (FC)|> 1.5, false discovery rate (FDR) < 0.05 and *P* < 0.05. All analyses were controlled for FDR by the Benjamini–Hochberg procedure. Gene Set Enrichment Analysis (GSEA) software was used for analysis, and the enrichment pathway was set to be significant based on the nominal (NOM) *P* < 0.05 and FDR *Q* < 0.05.

### Statistical analysis

Comparisons of continuous and categorical variables were performed using the Mann–Whitney’s U/Kruskal–Wallis test followed by Dunn’s post–hoc test and Pearson’s χ^2^/Fisher’s exact test, respectively. Both the Kaplan–Meier method (log-rank test) and Cox regression were used to analyze the intrinsic connection between *SPAG1* expression and survival time, including leukemia-free survival (LFS), event-free survival (EFS) and overall survival (OS). The receiver operating characteristic (ROC) curve and area under the ROC curve (AUC) were used to determine the discriminating ability of *SPAG1* expression for AML and controls. Two-sided *P* values < 0.05 in all statistical analyses were considered statistically significant.

## Results

### Identification of *SPAG1* among SPAG family members linked to AML prognosis in public datasets

**Fig. 1:**
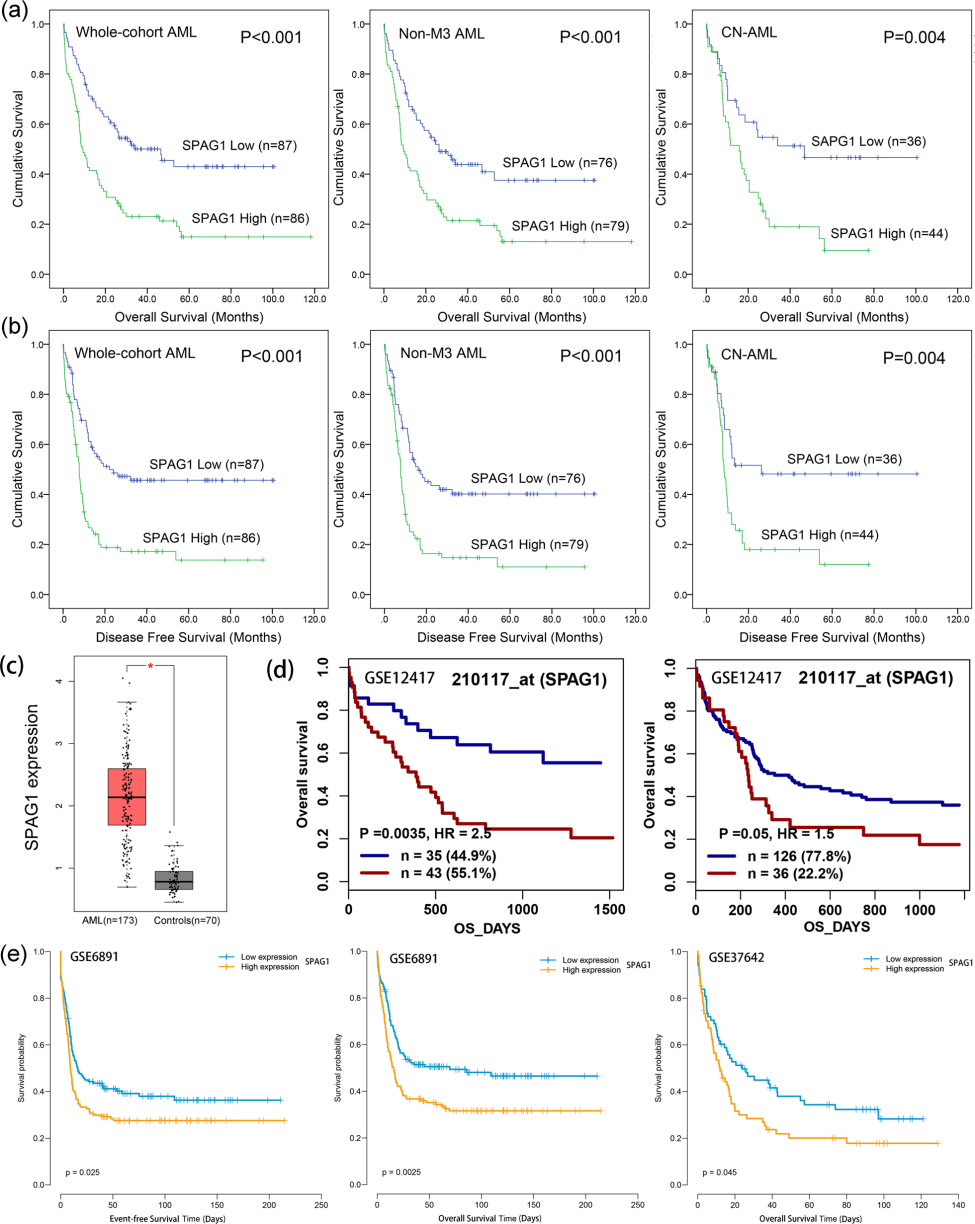
**The impact of *SPAG1* expression on survival of AML patients. a** The effect of *SPAG1* expression on overall survival in whole-cohort AML, non-M3 AML, and CN-AML from TCGA dataset. **b** The effect of *SPAG1* expression on disease/leukemia free survival in in whole-cohort AML, non-M3 AML, and CN-AML from TCGA dataset. **c** *SPAG1* expression in AML from TCGA dataset. **d** The effect of *SPAG1* expression on overall survival in 78 and 162 CN-AML from the GEO dataset (GSE12417) analyzed by the online web tool Genomicscape (http://genomicscape.com/microarray/survival.php). **e** The effect of *SPAG1* expression on event-free survival and overall survival in AML from GEO datasets (GSE6891 and GSE37642)

To explore the prognostic significance of the *SPAG* family members (*SPAG1/2/3/4/5/6/7/9/10/13/16/17*) in AML, we first determined the impact of each *SPAG* member on survival time (both OS and LFS) by Cox regression univariate analysis among AML in TCGA datasets. When analyzing the prognostic value of the *SPAG* family members in AML patients, each one was evaluated according to the difference between two groups of patients, divided by the median level of *SPAG* expression as the cutoff. As presented in Table 1 and Additional file 1: Table S2, only *SPAG1* expression had a significant connection with OS and LFS in both AML (both *P* < 0.001) and non-M3 AML (both *P* < 0.001) as well as CN-AML (*P* = 0.005 and 0.006, respectively). Furthermore, Kaplan–Meier analysis also revealed that AML patients with higher *SPAG1* expression showed significantly shorter OS and LFS than those with lower *SPAG1* expression among AML (both *P* < 0.001), non-M3 AML (both *P* < 0.001), and CN-AML (both *P* = 0.004) patients (Fig. 1a and b). In addition, the expression of *SPAG1* was upregulated in AML patients, as analyzed by GEPIA (Fig. 1c).

**Table 1:** Cox regression univariate analysis of variables for overall survival in AML patients

Variables	Whole-cohort AML		Non-M3 AML		CN-AML	
	HR (95% CI)	*P*	HR (95% CI)	*P*	HR (95% CI)	*P*
*SPAG1* expression	2.280 (1.562–3.327)	0.000	2.011 (1.366–2.962)	0.000	2.279 (1.283–4.047)	0.005
*SPAG2*/*UAP1* expression	1.313 (0.908–1.897)	0.148	1.536 (1.052–2.241)	0.026	1.051 (0.614–1.798)	0.857
*SPAG3*/*SPAG8* expression	0.901 (0.624–1.302)	0.578	1.021 (0.699–1.490)	0.914	0.801 (0.469–1.367)	0.416
*SPAG4* expression	0.881 (0.610–1.273)	0.501	1.047 (0.718–1.527)	0.812	0.762 (0.442–1.315	0.329
*SPAG5* expression	0.845 (0.584–1.223)	0.373	0.747 (0.509–1.096)	0.136	0.564 (0.319–0.998)	0.049
*SPAG6* expression	1.504 (1.039–2.178)	0.031	1.359 (0.929–1.988)	0.114	0.990 (0.575–1.705)	0.971
*SPAG7* expression	0.752 (0.519–1.089)	0.131	0.820 (0.560–1.202)	0.310	0.854 (0.498–1.467)	0.568
*SPAG9* expression	0.986 (0.683–1.423)	0.939	0.906 (0.621–1.321)	0.607	1.176 (0.686–2.014)	0.556
*SPAG10*/*MFGE8* expression	0.890 (0.616–1.287)	0.537	1.026 (0.703–1.498)	0.892	0.696 (0.406–1.193)	0.187
*SPAG13*/*ITPRID2* expression	0.854 (0.591–1.234)	0.401	0.777 (0.532–1.134)	0.191	1.170 (0.683–2.004)	0.567
*SPAG16* expression	1.427 (0.985–2.069)	0.060	1.257 (0.857–1.843)	0.242	1.320 (0.769–2.266)	0.314
*SPAG17* expression	0.936 (0.648–1.351)	0.722	0.913 (0.626–1.332)	0.637	0.850 (0.498–1.453)	0.553

AML: acute myeloid leukemia; CN-AML: cytogenetically normal AML; HR: hazard ratio; CI: confidence interval. The prognostic value of *SPAG11A*, *SPAG11B*, and *SPAG16/SPAM1* expression was not determined because the three members were hardly expressed in AML patients

Next, the prognostic value of *SPAG1* expression in AML was further validated in GEO datasets including GSE12417, GSE6891 and GSE37642. For GSE12417, the online platform GenomicScape (http://genomicscape.com/microarray/survival.php) also confirmed the prognostic correlation of *SPAG1* expression with OS in patients with CN-AML among two independent cohorts (*P* = 0.0035 and 0.05, respectively, Fig. 1d). For GSE6891 and GSE37642, Kaplan–Meier analysis showed that AML patients with higher *SPAG1* expression had strikingly shorter EFS and/or OS times than those with lower *SPAG1* expression (*P* = 0.025, 0.0025 and 0.045, respectively, Fig. 1e).

### Clinical implications of *SPAG1* expression in AML in the TCGA dataset

**Fig. 2:**
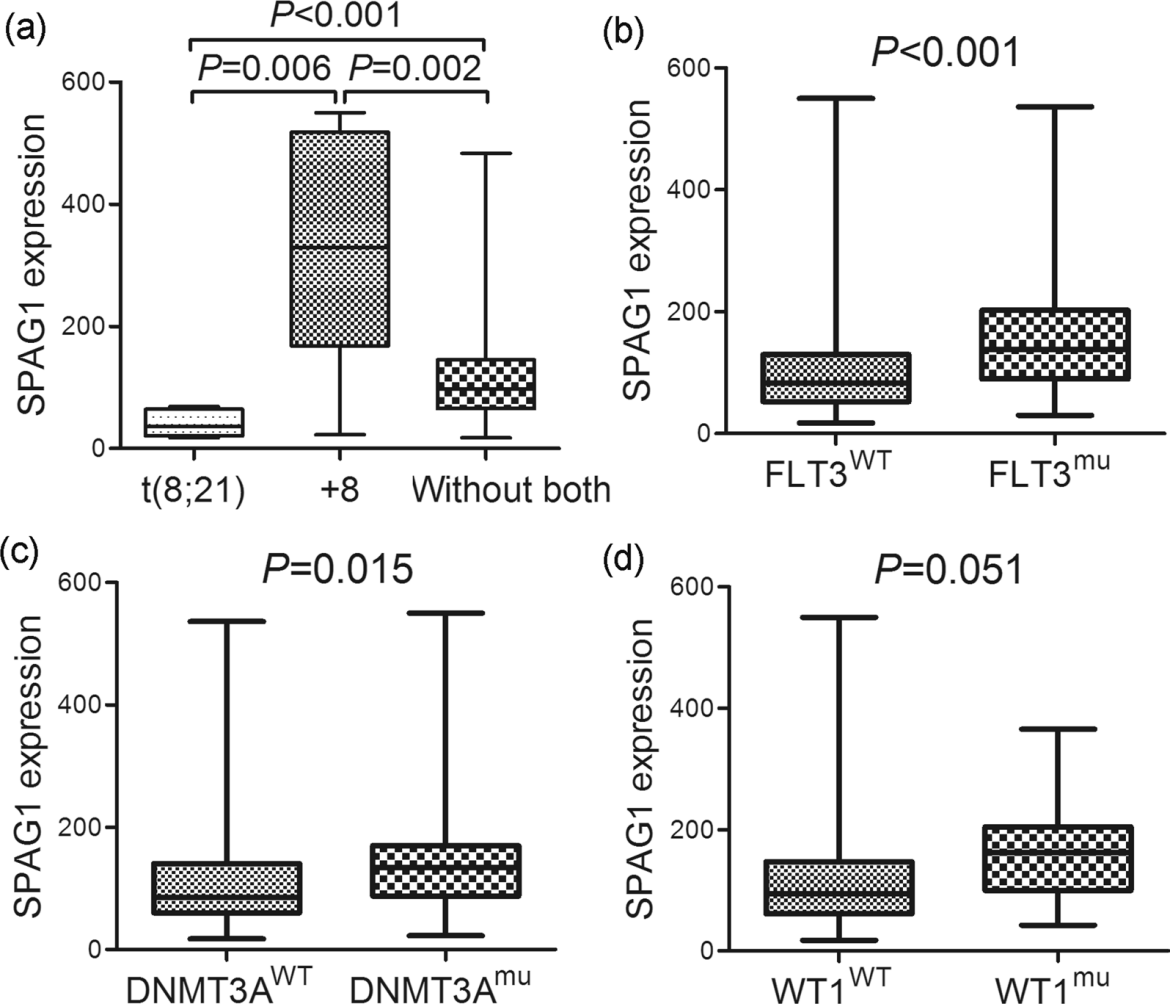
The associations of *SPAG1* expression with genetic abnormalities in AML. **a** *SPAG1* expression in AML patients with and without chromosome 8 abnormalities from TCGA datasets. **b** *SPAG1* expression in AML patients with and without *FLT3* mutations from TCGA datasets. **c** *SPAG1* expression in AML patients with and without *DNMT3A* mutations from TCGA datasets. **d** *SPAG1* expression in AML patients with and without *WT1* mutations from TCGA datasets

*SPAG1* was the only remaining *SPAG* member linked to AML prognosis, which prompted us to analyze the associations of *SPAG1* expression with the clinical/biological characteristics of AML patients. The differences between the high and low *SPAG1* groups in terms of sex, age, white blood cell (WBC) counts, peripheral blood (PB)/BM blasts, FAB classifications, cytogenetics, and gene mutations are shown in Table 2. Notably, cases with higher *SPAG1* expression had markedly higher WBC counts than did those with lower *SPAG1* expression (*P* = 0.014, Table 2). Furthermore, there were marked differences between the two groups regarding the occurrence rate of each FAB classification and cytogenetics (*P* = 0.024, Table 2). Cases with higher *SPAG1* expression were commonly classified as FAB-M4/M5 (*P* = 0.058 and 0.050, respectively, Table 2). Regarding cytogenetics, patients with higher *SPAG1* expression more commonly exhibited + 8 (*P* = 0.034) and rarely t(8;21) (*P* = 0.014, Table 2). We further showed *SPAG1* expression among groups with + 8, t(8;21) or neither (Fig. 2a). In addition, we revealed the associations of *SPAG1* expression with several of the most frequent gene mutations in AML (Table 2). Higher *SPAG1* expression was markedly or nearly correlated with *FLT3*, *DNMT3A*, and *WT1* mutations (*P* < 0.001, = 0.001 and = 0.057, respectively, Table 2). Moreover, we further compared *SPAG1* expression between patients carrying or not carrying these gene mutations and observed statistical significance in subgroups divided by *FLT3* and *DNMT3A* status (*P* < 0.001 and = 0.015, respectively, Fig. 2b and 2c), whereas a trend was observed in subgroups divided by *WT1* status (*P* = 0.051, Fig. 2d).

**Table 2:** Comparative analysis of *SPAG1* expression with clinic-pathologic characteristics in AML

Patient's parameters	*SPAG1* expression		
	Low (n = 87)	High (n = 86)	*P* value
Sex, male/female	46/41	46/40	1.000
Median age, years (range)	57 (18–88)	61 (21–81)	0.409
Median WBC, × 10^9^/L (range)	11 (0.6–297.4)	27.65 (0.4–223.8)	0.014
Median PB blasts, % (range)	41 (0–98)	36 (0–97)	0.544
Median BM blasts, % (range)	70 (32–100)	75 (30–98)	0.377
FAB classifications			0.024
M0	7	9	NS
M1	27	17	NS
M2	21	17	NS
M3	11	5	NS
M4	12	22	0.058
M5	5	13	0.050
M6	1	1	NS
M7	3	0	NS
No data	0	2	NS
Cytogenetics			0.006
Normal	36	44	NS
t(15;17)	10	5	NS
t(8;21)	7	0	0.014
inv(16)	8	2	NS
+8	1	7	0.034
del(5)	1	0	NS
−7/del(7)	4	3	NS
11q23	0	3	NS
Others	7	7	NS
Complex	11	14	NS
No data	2	1	NS
Gene mutation			
FLT3 (±)	14/73	35/51	0.000
NPM1 (±)	20/67	28/58	0.177
DNMT3A (±)	12/75	30/56	0.001
IDH2 (±)	10/77	7/79	0.611
IDH1 (±)	9/78	7/79	0.794
TET2 (±)	9/78	6/80	0.590
RUNX1 (±)	8/79	7/79	1.000
TP53 (±)	6/81	8/78	0.590
NRAS (±)	6/81	6/80	1.000
CEBPA (±)	6/81	7/79	0.782
WT1 (±)	2/85	8/78	0.057
PTPN11 (±)	4/83	4/82	1.000
KIT (±)	6/81	1/85	0.117
U2AF1 (±)	4/83	3/83	1.000
KRAS (±)	2/85	5/81	0.278

AML: acute myeloid leukemia; WBC: white blood cell; PB: peripheral blood; BM: bone marrow; FAB: French-American-British; NS: no significance

### Further confirmation of the prognostic value of *SPAG1* expression in AML in the TCGA dataset

Since a significant relationship was observed between *SPAG1* expression and some common prognostic factors such as WBC, cytogenetics and gene mutations, we performed multivariate analysis by Cox regression to confirm the effect of *SPAG1* expression on survival rate and demonstrated that *SPAG1* expression acted as a positive independent risk factor affecting OS and LFS in whole-cohort AML (both *P* < 0.001), non-M3 AML (*P* = 0.003 and 0.005, respectively), or CN-AML patients (*P* = 0.001 and 0.007, respectively) (Table 3 and Additional file 1: Table S3).

**Table 3:** Cox regression multivariate analysis of variables for overall survival in AML patients

Variables	Whole-cohort AML		Non-M3 AML		CN-AML	
	HR (95% CI)	*P*	HR (95% CI)	*P*	HR (95% CI)	*P*
Age	1.032 (1.016–1.047)	0.000	1.024 (1.008–1.041)	0.003	1.031 (1.012–1.051)	0.001
WBC	1.006 (1.001–1.010)	0.008	1.005 (1.000–1.009)	0.041	1.006 (1.001–1.011)	0.015
Molecular risks	2.112 (1.568–2.845)	0.000	2.101 (1.526–2.892)	0.000	1.769 (0.664–4.714)	0.254
Treatment regimen	0.421 (0.273–0.650)	0.000	0.389 (0.250–0.606)	0.000	0.614 (0.326–1.155)	0.131
*SPAG1* expression	2.162 (1.465–3.191)	0.000	2.048 (1.377–3.047)	0.000	2.419 (1.354–4.320)	0.003

AML: acute myeloid leukemia; CN-AML: cytogenetically normal AML; WBC: white blood cell. Variables including age (continuous variables), WBC (continuous variables), treatment regimen (with transplantation vs. without transplantation) and molecular risks (good, intermediate, poor, and unknown)

**Fig. 3:**
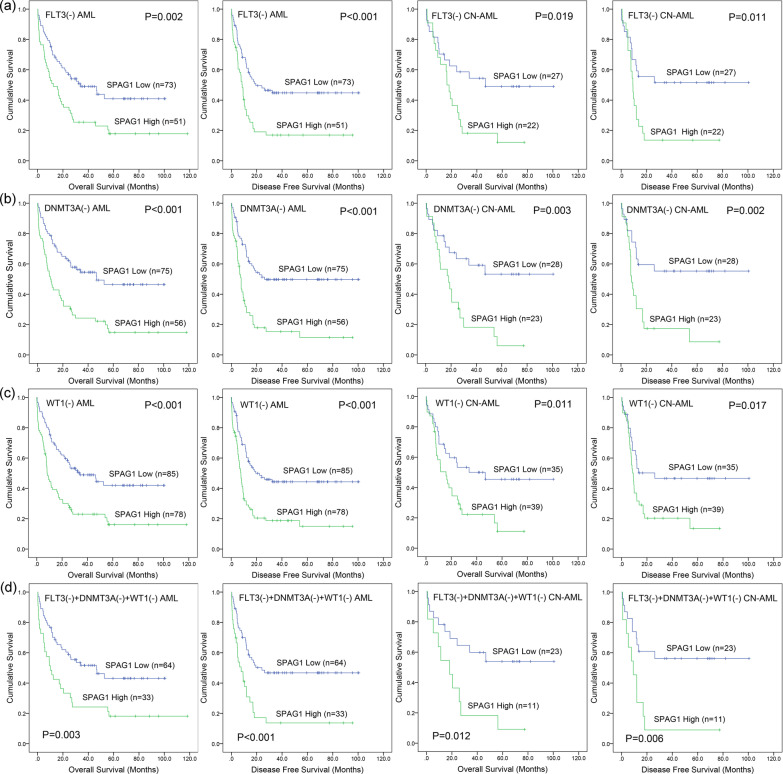
The impact of *SPAG1* expression on survival of AML patients with specific subtypes. **a** Kaplan–Meier survival curves of overall survival and disease/leukemia free survival in whole-cohort AML and CN-AML without *FLT3* mutation from TCGA datasets. **b** Kaplan–Meier survival curves of overall survival and disease/leukemia free survival in whole-cohort AML and CN-AML without *DNMT3A* mutation from TCGA datasets. **c** Kaplan–Meier survival curves of overall survival and disease/leukemia free survival in whole-cohort AML and CN-AML without *WT1* mutation from TCGA datasets. **d** Kaplan–Meier survival curves of overall survival and disease/leukemia free survival in whole-cohort AML and CN-AML without *FLT3*/*DNMT3A*/*WT1* mutation from TCGA datasets

Mutations in *FLT3*, *DNMT3A* and *WT1* are widely accepted factors that influence AML prognosis [2, 3]. According to this study, since *SPAG1* expression was significantly or nearly significantly correlated with *FLT3*, *DNMT3A* and *WT1* mutations, we further investigated the prognostic value of *SPAG1* expression in AML independent of these gene mutations. As Fig. 3 shows, both AML and CN-AML patients with higher *SPAG1* expression also exhibited markedly shorter OS and LFS times than those with poor *SPAG1* expression, regardless of the mutation status of *FLT3* (Fig. 3a), *DNMT3A* (Fig. 3b), *WT1* (Fig. 3c) or all the three genes (Fig. 3d).

### *SPAG1* expression is a prognostic indicator for AML after HSCT in the TCGA dataset and may have a guiding effect on treatment choice between chemotherapy and HSCT

**Fig. 4:**
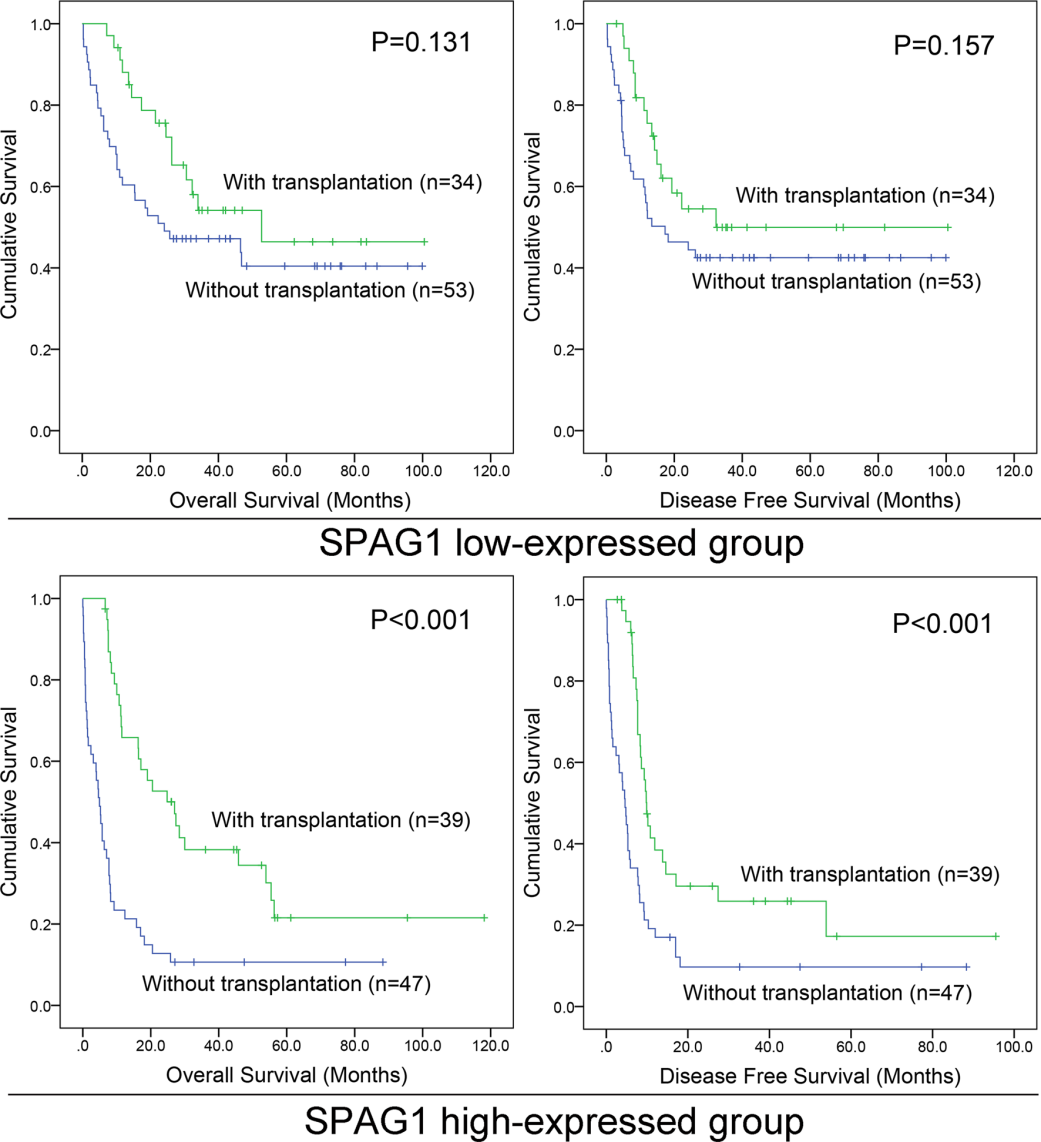
The effect of HSCT on survival of AML patients among different *SPAG1* expression groups. Kaplan–Meier survival curves of overall survival and disease/leukemia free survival among whole-cohort AML in both lower and higher *SPAG1* expression group from TCGA datasets

HSCT is an important consolidation treatment regimen in against disease recurrence in AML. To explore whether HSCT could nullify the negative prognostic effect of higher *SPAG1* expression in AML, we analyzed the effect of HSCT intervention on prognosis in both the lower and higher *SPAG1* expression groups. In the *SPAG1* overexpression group, HSCT for AML patients undergoing induction therapy markedly improved OS and LFS, which was not observed for those just receiving chemotherapy (both *P* < 0.001, Fig. 4). However, there were no obvious differences regarding OS and LFS between the HSCT and chemotherapy sets in the group of AML patients with lower *SPAG1* expression (*P* = 0.131 and 157, respectively, Fig. 4). To sum up the results, AML patients with *SPAG1* hyperexpression may profit from HSCT, which suggests that *SPAG1* expression may be used to guide therapeutic selection between HSCT and chemotherapy in AML patients undergoing induction therapy.

### Molecular signatures associated with *SPAG1* expression in AML in the TCGA dataset

**Fig. 5:**
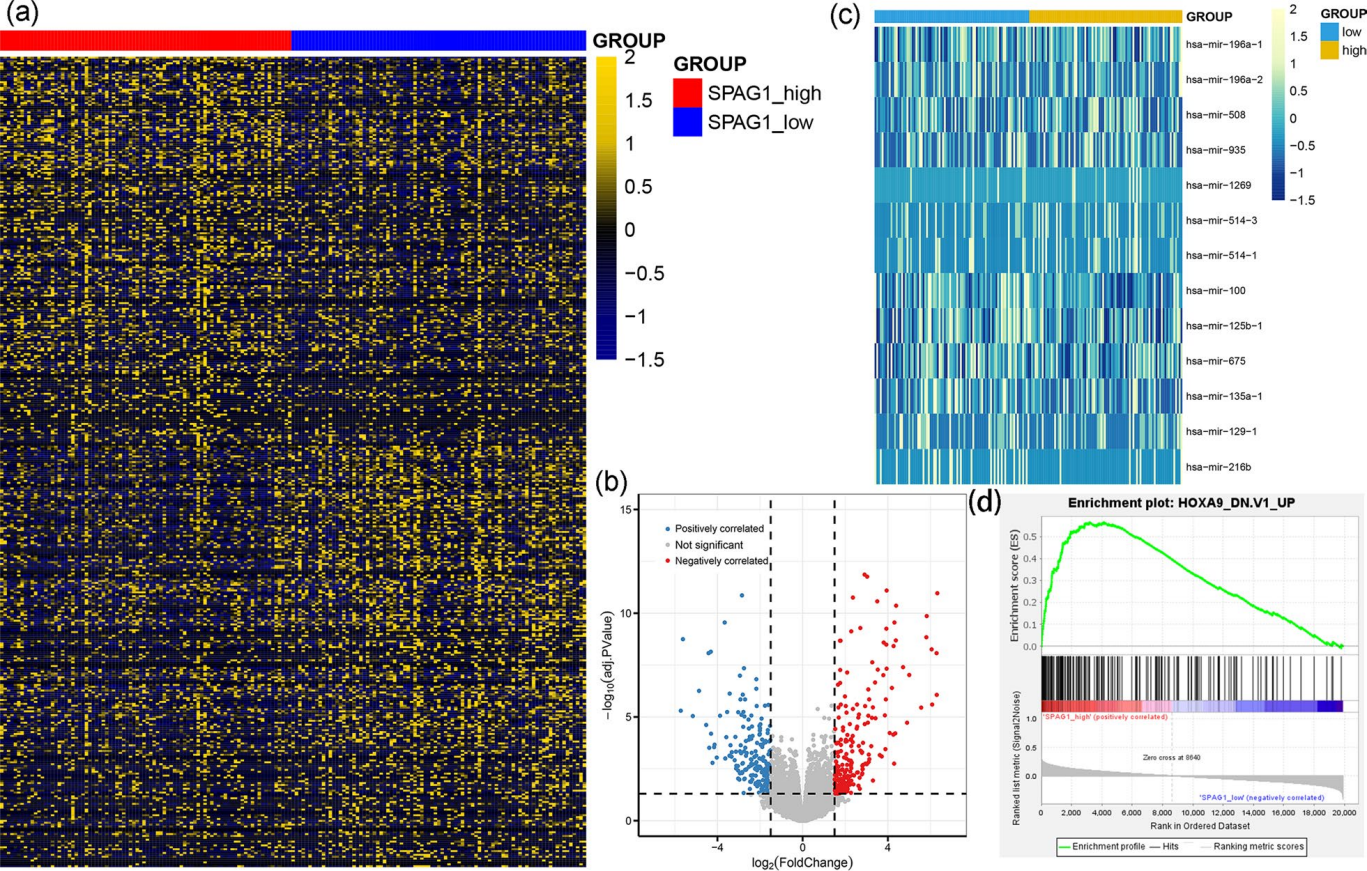
Molecular signatures associated with *SPAG1* in AML. **a** Expression heatmap of differentially expressed genes between AML patients with lower and higher *SPAG1* expression groups among TCGA datasets (FDR < 0.05, *P* < 0.05 and |log2 FC|> 1.5). **b** Volcano plot of differentially expressed genes between AML patients with lower and higher *SPAG1* expression (FDR < 0.05, *P* < 0.05, and |log2 FC|> 1.5). **c** Expression heatmap of differentially expressed microRNAs between AML patients with lower and higher *SPAG1* expression (FDR < 0.05, *P* < 0.05, and |log2 FC|> 1.5). **d** GSEA analysis of *SPAG1* expression associated with *HOXA9* dysregulation oin AML (NOM *P* < 0.05 and FDR *Q* < 0.05)

To explore the biological network in AML caused by abnormal *SPAG1* expression, we first compared the transcriptomes of AML samples with lower and higher *SPAG1* expression in the TCGA set. Up to 429 mRNAs and 13 miRNAs were found to be differentially expressed between two sets based on the following conditions: |log2 FC|> 1.5, FDR < 0.05 and *P* < 0.05 (Fig. 5a–c and Additional file 2: Table S4). Among these DEGs, 206 mRNAs and 7 miRNAs were found to be positively correlated with *SPAG1* expression, whereas 223 genes and 6 miRNAs were negatively correlated with *SPAG1* expression. Positively correlated genes such as *MECOM* were reported to have pro-leukemia effects [16] and were associated with prognosis in AML. Negatively correlated genes such as *RUNX1T1* and *LEP* were reported to have anti-leukemia effects and were also informative for AML prognosis [13]. Moreover, GSEA revealed that *SPAG1* might participate in *HOXA9* dysregulation associated with AML (Fig. 5d).

### Validation of *SPAG1* expression and its clinical significance in AML in our research cohort

**Fig. 6:**
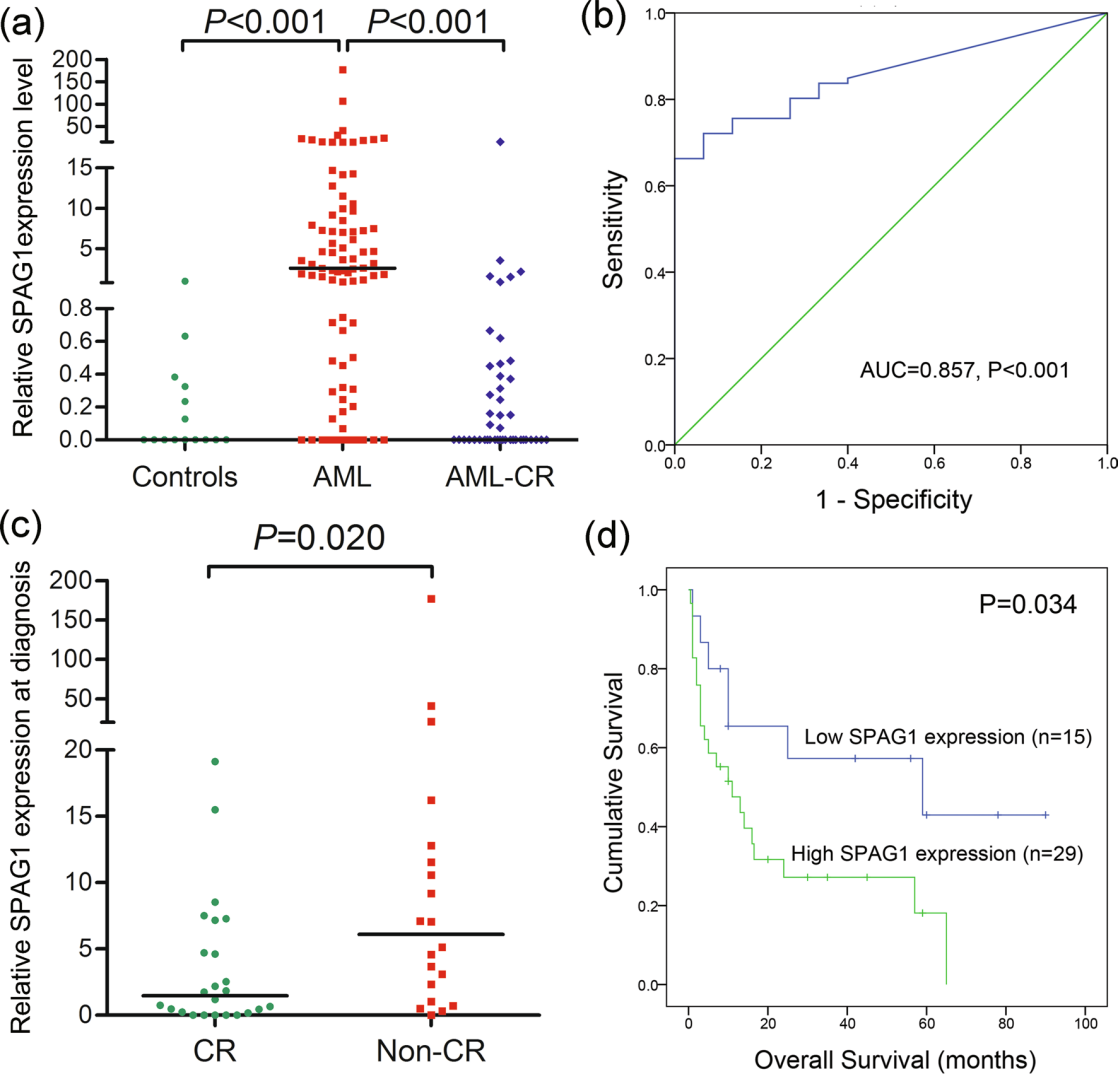
Validation of *SPAG1* expression and its clinical significance in AML. **a** *SPAG1* expression in 15 controls, 86 AML patients at diagnosis time, and 45 AML patients who achieved complete remission. **b** ROC curve analysis of *SPAG1* expression in distinguishing AML from controls. **c** *SPAG1* expression at diagnosis time in AML patients who did and did not achieve CR after 1–2 course induction therapy. **d** Kaplan–Meier survival curves of overall survival regarding *SPAG1* expression in whole-cohort AML from our hospital

To verify the expression pattern and clinical significance of *SPAG1* expression in AML, we further investigated *SPAG1* mRNA expression in BMMNC samples from 86 AML patients at diagnosis, 45 AML patients in the CR period and 15 healthy donors collected in our hospital. As expected, *SPAG1* expression was significantly increased in newly diagnosed AML patients compared with healthy controls and AML patients in CR (both *P* < 0.001, Fig. 6a). Moreover, ROC analysis revealed that *SPAG1* expression may serve as a quantifiable biomarker for distinguishing AML from controls, presenting an AUC of 0.857 (95% CI: 0.783–0.93) (*P* < 0.001, Fig. 6b). Significantly, AML patients who did not achieve CR after 1–2 courses of induction therapy exhibited markedly higher *SPAG1* expression levels at diagnosis than those who achieved CR after 1–2 courses of induction therapy (*P* = 0.020, Fig. 6c). According to the set point of 1.0198 determined by ROC analysis (sensitivity of 66.3% and specificity of 100%), we grouped AML patients into two sets to analyze the prognostic significance of *SPAG1* expression. Kaplan–Meier analysis demonstrated a marked tendency of shorter OS time in AML patients with high *SPAG1* expression than in those with low *SPAG1* expression (*P* = 0.034, Fig. 6d).

## Discussion

Recent evidence has characterized *SPAG* family member expression together with its functional roles in cancer development. For example, *SPAG1* expression could be enrolled in the early spread and adverse outcome of pancreatic adenocarcinoma and prostate cancer [17, 18]. *SPAG2*/*UAP1* has been shown to be a promising therapeutic target for bladder cancer as well as lung adenocarcinoma [19, 20]. *SPAG4* could act as a potential biomarker of glioblastoma progression and prognosis, as well as in renal cell carcinoma and lung carcinoma [21–23]. Moreover, *SPAG5* hyperexpression was connected to poor disease-free survival in breast cancer patients, and fueled breast cancer cell proliferation [24]. Interestingly, reduced expression of *SPAG6*, which is transcriptionally regulated by tumor specific DNA methylation, has been revealed in non-small-cell lung cancer [25]. A direct role for aberrant *SPAG9* was identified in diverse human cancers such as Kaposi’s sarcoma, gastric cancer, prostate cancer, thyroid carcinoma, liver cancer, and bladder transitional cell carcinoma [26–32]. Notably, accumulating studies have shown that *SPAG6* expression is correlated with the pathogenesis of myelodysplastic syndrome (MDS) and Burkett lymphoma (BL) [33–37]. Consequently, SPAG proteins serve as a novel type of CT antigen with contributions to cancer formation and are likely to be novel targets for tumor targeted therapies.

This study was the first to reveal *SPAG1* expression as uniquely associated with poor prognosis in AML among all *SPAG* family members through both analysis of public data and validation in a research cohort. It was demonstrated that *SPAG1* expression could be a promising prognostic biomarker and could be used to optimize the choice of therapy between chemotherapy and HSCT in AML. Unlike *SPAG6*, *SPAG1* expression has rarely been studied in hematological malignances. Conversely, several reports have examined at the relationships between *SPAG1* and solid tumors. Shamsara et al. demonstrated that the amplification of *SPAG1* was associated with decreased survival in patients with prostate cancer [18]. Moreover, *SPAG1* is an early expressed gene in pancreatic tumorigenesis and can promote the activity of cancer cells [17]. Lin et al. showed that *SPAG1* expression was a crucial variable related to many clinicopathological features and to RFS in breast cancer [38]. Functionally, *SPAG1* acts as an inhibitor of breast cancer cell proliferation and colony formation during breast cancer pathogenesis and development [38]. Since there was no deep insight into *SPAG1* in AML, further mechanistic studies are essential for investigating the possible role of *SPAG1* in leukemogenesis and AML development.

The current study also identified a significant association between *SPAG1* expression and genetic (both cytogenetic and molecular) abnormalities in AML. We first found the associations of *SPAG1* expression with FAB-M4/M5 disease, suggesting that *SPAG1* expression may play a role in monocyte differentiation and monocyte leukemogenesis. In terms of cytogenetics, *SPAG1* expression was positively correlated with +8 but negatively associated with t(8;21) (q22;q22). Since the *SPAG1* gene is located in 8q22.2, it is not surprising that aberrant *SPAG1* expression was associated with these chromosome abnormalities. Notably, further studies are needed to determine whether the functions of these chromosomal abnormalities during leukemogenesis occur through aberrant *SPAG1* expression. Regarding gene mutations, *SPAG1* expression was associated with *FLT3* and *DNMT3A* mutations in AML, but the exact relationship between *SPAG1* expression and these gene mutations still unclear. Importantly, there is no evidence showing the association of *FLT3* and *DNMT3A* mutations with the above chromosome abnormalities. Consequently, we need to obtain deeper insight into the underlying mechanism of *SPAG1* expression in leukemogenesis caused by *FLT3* and/or *DNMT3A* mutations.

In general, our discoveries suggested that *SPAG1* hyperexpression may function independently as a prognostic biomarker and assist treatment selection between HSCT and chemotherapy in AML.

## Supplementary Information

**Additional file 1:** Clinic-pathologic characteristics of AML patients in our research cohort and Cox regression univariate and multivariate analysis of variables for leukemia free survival in AML patients in TCGA dataset.

**Additional file 2:** Different expressed genes correlated with SPAG1 expression.

## Data Availability

The datasets used and/or analyzed during the current study are available from the corresponding author on reasonable request.
