# Supplementary material for: Comprehensive analysis of SPAG1 expression as a prognostic and predictive biomarker in acute myeloid leukemia by integrative bioinformatics and clinical validation
Source: BMC Med Genomics. 2022 Feb 28;15:38. doi: 10.1186/s12920-022-01193-0 (PMC8886923; doi:10.1186/s12920-022-01193-0)
Supplement: Supplementary file 1 — Additional file 1: Clinic-pathologic characteristics of AML patients in our research cohort and Cox regression univariate and multivariate analysis of variables for leukemia free survival in AML patients in TCGA dataset. [file 12920_2022_1193_MOESM1_ESM.docx]

**Supplementary Table S1. Clinic-pathologic characteristics of AML in our research cohort**

|  | Patient's parameters (n=86) |
| --- | --- |
| Sex, male/female | 48/38 |
| Median age, years (range) | 52 (18-81) |
| Median WBC, ×10^9^/L (range) | 27.8 (0.3-528.0) |
| Median hemoglobin, g/L (range) | 80 (42-131) |
| Median platelets, ×10^9^/L (range) | 34 (4-128) |
| BM blasts, % (range) | 43.5 (1.0*-97.5) |
| FAB subtypes |  |
| M0 | 2 |
| M1 | 5 |
| M2 | 30 |
| M3 | 18 |
| M4 | 15 |
| M5 | 14 |
| M6 | 4 |
| CR (-/+) | 45/41 |

AML: acute myeloid leukemia; WBC: white blood cells; BM: bone marrow; FAB: French-American-British classification; CR: complete remission. *: AML patients less than 20% BM blasts often with typical cytogenetics such as t(15;17).

**Supplementary Table S2. Cox regression univariate analysis of variables for leukemia free survival in AML patients**

| Variables | Whole-cohort AML | | Non-M3 AML | | CN-AML | |
| --- | --- | --- | --- | --- | --- | --- |
|  | HR (95% CI) | *P* | HR (95% CI) | *P* | HR (95% CI) | *P* |
| *SPAG1* expression | 2.442 (1.669-3.575) | 0.000 | 2.227 (1.506-3.292) | 0.000 | 2.243 (1.265-3.978) | 0.006 |
| *SPAG2*/*UAP1* expression | 1.366 (0.945-1.975) | 0.097 | 1.666 (1.140-2.434) | 0.008 | 1.210 (0.707-2.072) | 0.487 |
| *SPAG3*/*SPAG8* expression | 0.927 (0.641-1.338) | 0.684 | 1.056 (0.723-1.541) | 0.779 | 0.818 (0.479-1.396) | 0.462 |
| *SPAG4* expression | 0.858 (0.594-1.239) | 0.413 | 1.007 (0.691-1.469) | 0.970 | 0.747 (0.433-1.289) | 0.295 |
| *SPAG5* expression | 0.868 (0.601-1.253) | 0.449 | 0.779 (0.533-1.138) | 0.196 | 0.611 (0.350-1.067) | 0.083 |
| *SPAG6* expression | 1.588 (1.097-2.299) | 0.014 | 1.425 (0.974-2.085) | 0.068 | 1.059 (0.615-1.825) | 0.836 |
| *SPAG7* expression | 0.731 (0.505-1.058) | 0.097 | 0.783 (0.534-1.147) | 0.209 | 0.824 (0.481-1.411) | 0.481 |
| *SPAG9* expression | 0.996 (0.690-1.438) | 0.984 | 0.923 (0.633-1.346) | 0.676 | 1.192 (0.697-2.040) | 0.522 |
| *SPAG10*/*MFGE8* expression | 0.870 (0.602-1.259) | 0.461 | 1.013 (0.694-1.479) | 0.947 | 0.719 (0.420-1.233) | 0.231 |
| *SPAG13*/*ITPRID2* expression | 0.871 (0.603-1.258) | 0.462 | 0.805 (0.552-1.175) | 0.262 | 1.212 (0.707-2.076) | 0.485 |
| *SPAG16* expression | 1.388 (0.958-2.009) | 0.083 | 1.230 (0.839-1.802) | 0.288 | 1.322 (0.771-2.268) | 0.310 |
| *SPAG17* expression | 0.896 (0.620-1.294) | 0.557 | 0.876 (0.601-1.278) | 0.492 | 0.768 (0.449-1.312) | 0.334 |

AML: acute myeloid leukemia; CN-AML: cytogenetically normal AML; HR: hazard ratio; CI: confidence interval. The prognostic value of *SPAG11A*, *SPAG11B*, and *SPAG16/SPAM1* expression was not determined because the three members were hardly expressed in AML patients.

**Supplementary Table S3. Cox regression multivariate analysis of variables for leukemia free survival in AML patients**

| Variables | Whole-cohort AML | | Non-M3 AML | | CN-AML | |
| --- | --- | --- | --- | --- | --- | --- |
|  | HR (95% CI) | *P* | HR (95% CI) | *P* | HR (95% CI) | *P* |
| Age | 1.029 (1.014-1.044) | 0.000 | 1.022 (1.007-1.038) | 0.005 | 1.025 (1.007-1.044) | 0.007 |
| WBC | 1.006 (1.002-1.010) | 0.004 | 1.005 (1.001-1.009) | 0.018 | 1.006 (1.001-1.011) | 0.019 |
| Molecular risks | 1.860 (1.400-2.472) | 0.000 | 1.838 (1.357-2.490) | 0.000 | 1.252 (0.483-3.241) | 0.644 |
| Treatment regimen | 0.528 (0.348-0.802) | 0.003 | 0.498 (0.326-0.761) | 0.001 | 0.708 (0.378-1.326) | 0.281 |
| *SPAG1* expression | 2.109 (1.428-3.115) | 0.000 | 2.022 (1.357-3.013) | 0.001 | 2.185 (1.229-3.884) | 0.008 |

AML: acute myeloid leukemia; CN-AML: cytogenetically normal AML; WBC: white blood cells. Variables including age (continuous variables), WBC (continuous variables), treatment regimen (with transplantation vs. without transplantation) and molecular risks (good, intermediate, poor, and unknown).
